# ATP-triggered mitochondrial cascade reactions for cancer therapy with nanoscale zeolitic imidazole framework-90

**DOI:** 10.7150/thno.59593

**Published:** 2021-06-26

**Authors:** Wei Pan, Bingjie Cui, Kaiye Wang, Mingwan Shi, Fei Lu, Na Li, Bo Tang

**Affiliations:** College of Chemistry, Chemical Engineering and Materials Science, Key Laboratory of Molecular and Nano Probes, Ministry of Education, Collaborative Innovation Center of Functionalized Probes for Chemical Imaging in Universities of Shandong, Institute of Molecular and Nano Science, Shandong Normal University, Jinan 250014, P. R. China.

**Keywords:** zeolitic imidazole framework-90, 2-methoxyestradiol, camptothecine, reactive oxygen species, ATP

## Abstract

**Goals:** Chemotherapy, the most conventional modality for cancer therapy, usually brings serious side effects because of the low cancer-therapeutic specificity and bioavailability. It is of great significance for cancer treatment to develop new effective strategies to regulate biochemical reactions in organelles, enhance the specificity of chemotherapeutic drugs and reduce their side effects.

**Methods:** We report herein a zeolitic imidazole framework-90 (ZIF-90) based nanoplatform, which was used to initiate a series of mitochondrial cascade reactions using ATP as a molecular switch for cancer therapy. The thioketal linked camptothecin (camptothecin prodrug, TK-CPT) and 2-Methoxyestradiol (2-ME) were encapsulated into the pores of ZIF-90 nanoparticles using a simple one-pot method, and the nanoplatform was finally coated with a layer of homologous cell membrane.

**Results:** Mitochondrial ATP can efficiently degrade ZIF-90 and then release the loaded 2-ME and CPT prodrugs. 2-ME can inhibit the activity of superoxide dismutase (SOD), which induces the up-regulation of reactive oxygen species (ROS) *in situ*. The thioketal linkers in CPT prodrug can respond to ROS, thereby achieving subsequent release of parent CPT drug. This cascade of reactions can lead to prolonged high oxidative stress and cause continuous cancer cell apoptosis, due to the increased ROS level and the liberation of CPT.

**Conclusion:** We constructed an ATP-triggered strategy using nanoscale ZIF-90 to initiate mitochondrial cascade reactions for cancer therapy. The ZIF-90 based nanoplatform exhibited low cytotoxicity, good mitochondria-targeting ability, and excellent therapeutic effect. *In vivo* experiments demonstrated that the growth of tumor can be efficiently inhibited in a mouse model. This ATP-triggered strategy to induce mitochondrial biochemical reactions offers more possibilities for developing organelle-targeted therapeutic platforms.

## Introduction

Chemotherapy, the most conventional modality for cancer therapy, usually brings serious side effects because of the low cancer-therapeutic specificity and bioavailability 1, 2. To enhance the specificity of chemotherapy drugs and reduce their side effects, a prodrug strategy that responds to the tumor microenvironments has been invented and developed 3. For example, camptothecin (CPT) is a typical mitochondrial drug, which can work as an inhibitor for both cellular respiration and DNA topoisomerase I during cancer treatment 4. The CPT prodrug (thioketal-linked CPT, TK-CPT), formed by the covalent interaction of thioketone and CPT, can release the parent CPT under the stimuli of reactive oxygen species (ROS) 5. However, thioketal groups could only respond to the ROS at the millimolar level, which is obviously higher than the level of ROS in tumor cells 6, 7. In order to improve the level of ROS in mitochondria, remodeling of tumor redox microenvironment by generating ROS *in situ* or inhibiting the elimination of ROS in tumor tissue to increase the oxidative stress have been considered as a promising solution to effectively trigger the drug release 8, 9. 2-Methoxyestradiol (2-ME) has been proved to be able to elevate ROS level in tumor sites by inhibiting superoxide dismutase (SOD) 10, which is beneficial to cause oxidative stress and accelerate the release of parent CPT from the prodrug TK-CPT. Hence, how to deliver these drugs into mitochondria and regulate related biochemical reactions in a controlled manner is a key problem that needs to be solved urgently.

Zeolitic imidazole frameworks (ZIFs), as a subclass of metal-organic frameworks (MOFs), have attracted much attention due to their structural stability, tuneable pore sizes, and low toxicity 11-13. Among which, ZIF-90, synthesized with imidazolate-2-carboxyaldehyde and Zn^2+^, has been widely applied in the fields of biological detection and drug delivery 14-17. Recently, Mao et al. established a nanoplatform with nanoscale ZIF-90, which can image mitochondrial adenosine triphosphate (ATP) fluctuation in living cells 18. Because of the positive charges on the surface, ZIF-90 can actively target mitochondria via the electrostatic interaction 19, 20. Moreover, ZIF-90 can be decomposed by ATP in mitochondria to release fluorescent dyes for ATP imaging due to the competitive coordination between Zn^2+^ and ATP. Inspired by this idea, we consider that ATP can be employed as a molecular switch to control mitochondrial drug release and regulate related biochemical reactions in ZIF-90-based system. Such ATP-triggered strategy may be effective to enhance the efficiency of cancer therapy and reduce the side effect of drugs.

**Scheme 1:**
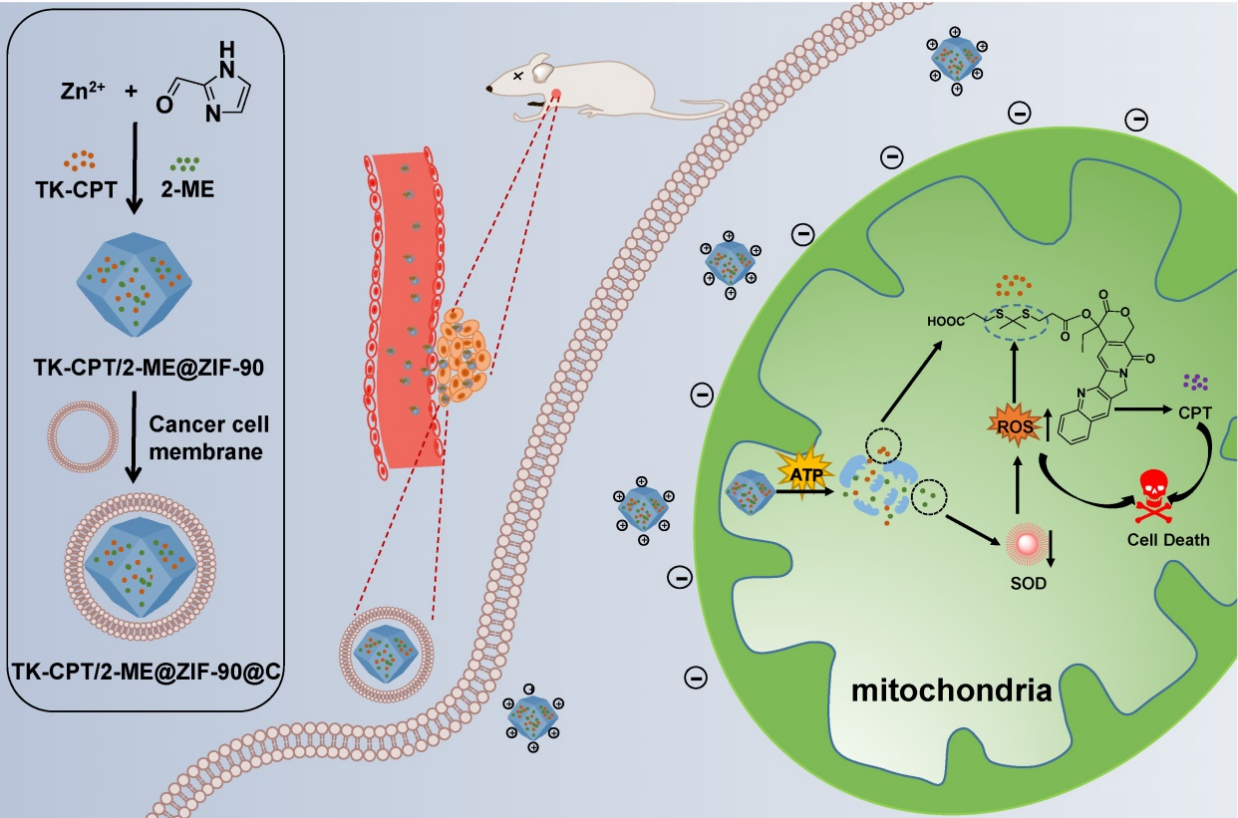
Schematic illustration of the preparation of the ZIF-90 based platform and cancer treatment processes.

In this study, an ATP-triggered strategy with nanoscale ZIF-90 to initiate mitochondrial cascade reactions for cancer therapy is proposed. 2-ME/TK-CPT@ZIF-90 (MTZ) nanoparticles were synthesized using a simple one-pot method, and coated with a layer of homologous cell membrane to target tumor tissues 21, 22. When MTZ nanoparticles entered mitochondria under the function of ZIF-90 surface charges, 2-ME inhibitor and TK-CPT can be released by the ATP-triggered decomposition. 2-ME can amplify the ROS level in mitochondria by inhibiting SOD, and therefore plays a vital role in tumors therapy by increasing tumor oxidative stress. With the increment of ROS level, the thioketal linkers in CPT prodrug can respond to incremental ROS and release the parent CPT drug. The ATP-triggered mitochondrial cascade reactions can cause high oxidative stress and induce cancer cells apoptosis. The detailed treatment processes are shown in Scheme 1.

## Methods

### Synthesis of ROS-cleavable thioketal linker (TK)

In a typical reaction, a mixture of anhydrous 3-mercaptopropionic acid (11.44 g, 108.02 mmol), anhydrous acetone (2.90 g, 49.10 mmol), and a catalytic amount of trifluoroacetic acid were stirred at 25 °C for 6 h. After the reaction, the flask was sealed and chilled in ice until the crystallization completed. The crystals were filtered, and washed with hexane and cold water. The product was obtained after dried in a vacuum desiccator.

### Synthesis of ROS-cleavable CPT prodrug (TK-CPT)

Briefly, a mixture of CPT (418.0 mg, 1.2 mmol), TK (604.9 mg, 1.2 mmol) and 2,2-bis(hydroxymethyl) propionic acid (DMAP) (124.4 mg, 1.0 mmol) were suspended in anhydrous dichloromethane (DCM) (45 mL), and then carbodiimide (EDC) (1150.2 mg, 6.0 mmol) was added into the mixed solution. After that, the reaction was stirred for 6 h at 25 °C, and a clear solution in yellow was finally obtained. The reaction mixture was extracted using brine for several times, and the obtained organic layer was dried over anhydrous Na_2_SO_4_. After removal of the solvent using a rotary evaporator, crude product as a yellow solid was achieved. The resulted solid residues were purified by column chromatography using petroleum ether/ethyl acetate as an eluent to afford TK-CPT.

### Synthesis of ZIF-90

Imidazole-2-formaldehyde (ICA, 40.00 mg, 0.42 mmol) was added into N,N-Dimethyl formamide (DMF) (9 mL). The solution was heated to 50 °C under stir until ICA fully dissolved, and then cooled to room temperature. Zn(COOH)_2_·6H_2_O (33.00 mg, 0.15 mmol) solution in DMF (1 mL)was added dropwise into the above ICA solution. The mixture was stirred for 10 min, and then trioctylamine (TOA, 500 µL) was added. After stirring at 25 °C for 12 h, the reaction was stopped. Particles were then isolated by centrifugation at 12000 revolutions per minute (rpm) for 10 min. After completely washed using DMF and EtOH for one and two times, respectively, the product was dried at 40 °C to obtain ZIF-90 nanoparticles.

### Synthesis of 2-ME/TK-CPT@ZIF-90 (MTZ)

ICA (40.00 mg, 0.42 mmol) dissolved in DMF (9 mL) was heated at 50 °C under stirring, until the solid was fully dissolved, and then cooled it to room temperature. 2-ME (10.00 mg) and TK-CPT (20.00 mg) were dissolved into the cooled solution. Zn(COOH)_2_·6H_2_O (33.00 mg, 0.15 mmol) in DMF (1 mL) was added dropwise into the ICA solution, and the mixture was stirred for 10 min. After that, TOA (500 µL) was added, and the reaction was stirred for 12 h. Particles were then isolated by centrifugation at 12000 rpm for 10 min. After completely washed using DMF and EtOH for one and two times, respectively, the product was dried at 40 °C to obtain MTZ nanoparticles (45.20 mg).

### Synthesis of 2-ME/TK-CPT@ZIF-90@C (MTZ@C)

First, the cells are digested and centrifuged for precipitation. The precipitated cells were washed twice using Tris-MgCl_2_ buffer (pH = 7.4), and resuspended with tris buffer containing protease inhibitor. The cells were broken by homogenizer every 2 min for duration of 18 s with totally 8 times. The whole process was performed in an ice-water bath. The cell membrane fragments were obtained by differential centrifugation of the broken cells. The cell membrane fragments were achieved by first centrifuging the broken cells at 500 rpm for 10 min, discarding the precipitate and retaining the supernatant. Then the supernatant was centrifuged for another 10 min at a speed of 10000 rpm, and the precipitate and supernatant parts were discarded and taken, respectively. The resulted supernatant was mixed with nanomaterials and stirred at a low temperature for 24 h with a slow speed. The final membrane coating material was successfully obtained through centrifugation.

### pH effect on the stability of MTZ nanoparticles

MTZ nanoparticles (1 mg/mL, 1 mL) were dispersed in the aqueous solutions with different pH values (4.3, 6.5, 7.4, and 8.0). After incubated for 0 and 2 h, the emission spectra of the above solutions using the supernatant part were detected to determine the amount of free TK-CPT, using the excitation wavelength of 380 nm. The concentrations of free TK-CPT were calculated through the intensity at 430 nm in the emission spectra. The degradation rates of MTZ in different pH values were then calculated according to the loading of TK-CPT at different times.

### ROS-responsive drug release

The CPT drug release was investigated by incubating TK-CPT (1 mg/mL) with O_2_^-^. O_2_^-^ was generated by potassium superoxide (KO_2_) dissolved in DMSO. The ROS-responsive behaviour of TK-CPT was studied using HPLC analysis. Solutions of CPT, TK-CPT and ROS + TK-CPT in water (1 mg/mL) were prepared and added to an equal volume of DMSO containing 1 mM O_2_^-^. After 24 h, 20 µL of each solution was taken for HPLC analysis.

### Cell culture

Mouse breast cancer cell line (4T1 cells) and mouse lung epithelial cell line (TC-1 cells) were treated with 1640 containing 10% fetal bovine serum and 1% 100 U/mL penicillin/streptomycin and were incubated under 37 °C. Anaerobic culture condition was 5% CO_2_, 1% O_2_ and 94% N_2_ at 37 °C.

### *In vitro* endocytic pathways

Endocytic pathways: First, 4T1 cells were divided into 4 groups: pre-treated with chlorpromazine (CPZ, 20 µM, incubation for 1 h, inhibitor of clathrin-mediated endocytsis), ethylisopropylamiloride (EIPA, 50 µM, incubation for 1 h, inhibitor of macropinocytosis), low temperature (4 °C, incubation for 2 h, energy inhibitor), and no inhibitor at 37 °C (control group). Then, the cells were washed with PBS for three times and cultured with MTZ@C (25 μg/mL) for 2 h at 37 °C. Finally, the cells were washed with PBS for three times and visualized under confocal fluorescence microscopy with 380 nm excitation. The fluorescence signal intensity of TK-CPT was analysed with the Image J software to evaluate the cellular MTZ@C.

### MTT assay

4T1 cells were inoculated into 96-well plates and stayed overnight to stick to the wall. The cells were divided into 5 groups (PBS, ZIF-90@C, TZ@C, MZ@C and MTZ@C), and each tested agent was diluted with culture medium to different concentrations (5, 10, 15, 20 and 25 μg/mL). The tested agents were added to the 96-well plates and incubated with cells for 10 h. Then the medium was removed from the wells, and 150 µL of MTT solution (0.5 mg/mL) was added for incubation for another 4 h. Finally, the MTT was removed, and 150 µL of DMSO was added. The absorbance of each group at 490 nm was measured by a microplate reader.

The cytotoxicity of MTZ@C under the condition of reduced intracellular concentration of ATP was studied by pre-treating 4T1 cells with 15 mM NaN_3_ for 12 h before incubated with MTZ@C. The MTZ@C nanoparticles were added to the 96-well plates and incubated with cells for 10 h. Then the medium was removed from the wells, and 150 µL of MTT solution (0.5 mg/mL) was added and incubated for another 4 h. Finally, the MTT solution was removed, and 150 µL of DMSO was added. The absorbance of each group at 490 nm was measured by a microplate reader.

### Live and dead cell staining assay

4T1 cells were inoculated into confocal dishes and divided into 5 groups (PBS, ZIF-90@C, TZ@C, MZ@C and MTZ@C). The cells were cultured overnight to stick to the wall before adding the materials. Then, the five groups of cells were incubated with the above materials for 10 h. The mediums containing different materials were removed. After washing twice with PBS, Calcein-AM/PI probe was added to the confocal dishes and incubated for another 30 min, and the death stain was then added. Finally, the cells were washed twice using PBS, and confocal imaging was performed using a TCS SP8 confocal microscope.

### Colocalization assay

Confocal laser-scanning microscope (CLSM) imaging was performed to observe the localization of MTZ@C in cells at different intervals. Briefly, 4T1 cells were seeded in chambered coverslips at a density of 3 × 10^5^ cells. After incubation for 24 h, the cells were incubated with MTZ@C for different times (0.5, 1.0, 1.5, 2.0 h). Cells were washed twice with PBS, and then incubated with mitotracker green to label mitochondria according to the manufacturer protocol. MTZ@C was labelled by the fluorescence of TK-CPT (*λ*_em_ = 380, *λ*_mx_ = 430), and the cells were imaged by the CLSM imaging system with identical settings.

### Detection of intracellular reactive oxygen species (ROS)

First, the cells were divided into five groups (PBS, ZIF-90@C, TZ@C, MZ@C and MTZ@C) and incubated overnight to adhere to the wall. Then, the cells were incubated with the different materials for 3 h. The probe 2',7'-dichlorodihydrofluorescein diacetate (DCFH-DA) was added to the five groups of cells, and incubated for 15 min. The cells were washed with PBS for 3 times and observed through CLSM.

### Tumor model establishment

All procedures of animal study were approved by Principles of Laboratory Animal Care (People's Republic of China) and the Guidelines of the Animal Investigation Committee, Biology Institute of Shandong Academy of Science, China. Female BALB/c mice (6-8 weeks) were housed under normal conditions. 4T1 cells were suspended in 50 µL 1640 media and subcutaneously injected into the alar of the mice. The tumor volume was calculated by the formula: volume = length × width^2^/2. The mice were treated when the tumor volumes were about 70 mm^3^.

### *In vivo* tumor targeting assay

#### ICP-AES

4T1 tumor mice were divided into two groups, one group was intravenous injected with MTZ NPs, and the other group was intravenous injected with MTZ@C NPs. After 24 h, the mice metabolites (the volume of mice urine was finally fixed to 10 mL) were collected and dissected. The organs and tumor tissues were separately dissolved in water for ICP tests. The concentration of zinc was measured by the following equation:

ID = ICP value/material injection quality/organ quality × 100%

#### Fluorescence imaging

ICG-loaded ZIF-90-ICG and ZIF-90@C-ICG were synthesized using the following protocols: 1 mg ZIF-90 NPs were dispersed into 2 mL aqueous solution and 20 µL of ICG solution (10 mg/mL) was added into the solution. The solution was stirred for 12 h and the product of ICG-loaded ZIF-90 NPs were obtained after centrifugation. Finally, the membrane of 4T1 cancer cells was encapsulated on the surface of ZIF-90-ICG to obtain ZIF-90@C-ICG.

50 µL of 4 mg/mL ICG-loaded ZIF-90-ICG and ZIF-90@C-ICG NPs were injected into the tumors of mice. At different time points of 0, 6, 12, 24, 36 h after injection, the fluorescence intensity of the mice were recorded by a live body imaging system.

### *In vivo* antitumor therapy

The tumor-bearing mice were divided into 5 groups, and injected intravenously with PBS, ZIF-90@C, TZ@C, MZ@C and MTZ@C (10 mg/kg), respectively. After two days, the tumor-bearing mice in the five groups were injected intravenously again with the same materials mentioned above. Tumor volume and body weights were recorded every 2 days and lasted for 2 weeks. The H&E staining of the organs (heart, liver, spleen, lung, and kidney) were tested at 7 days post-injection and the volumes of tumors were measured after 12 h of each treatment.

## Results and Discussion

### Synthesis and characterization of ZIF-90 based nanoplatform

**Figure 1:**
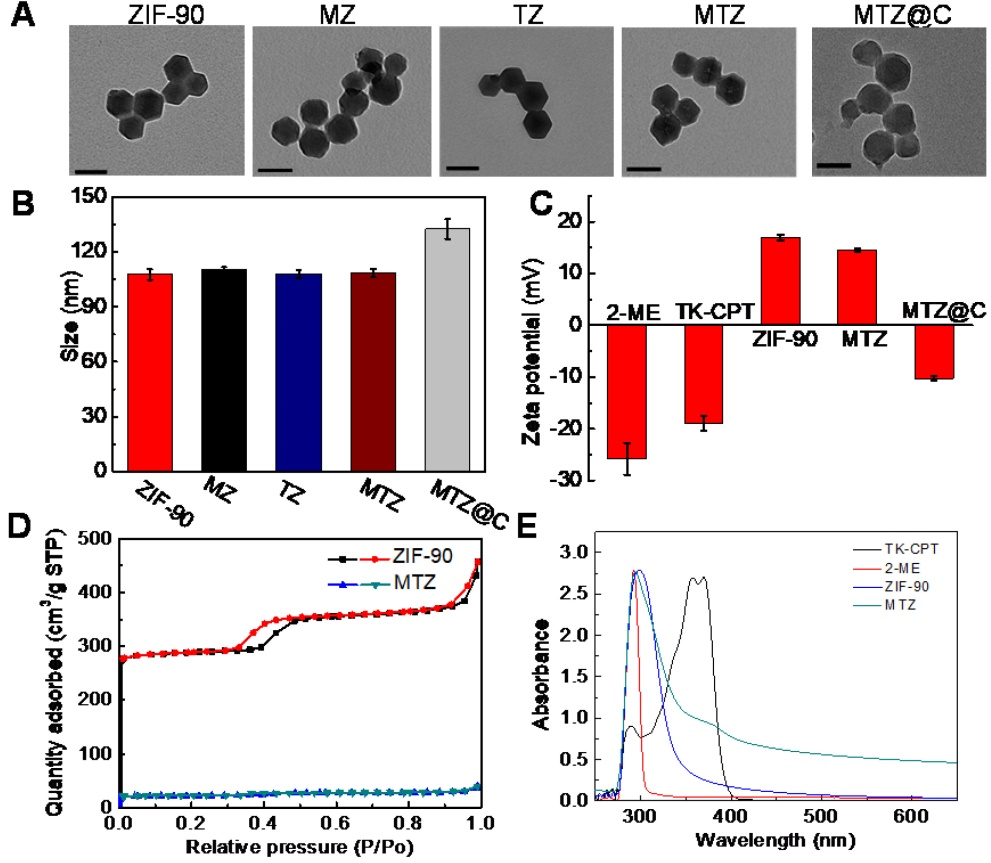
** (A)** TEM images of ZIF-90, MZ, TZ, MTZ, and MTZ@C. Scale bars are 100 nm. **(B)** Hydrodynamic size distributions of different nanoparticles. **(C)** Zeta potentials of different nanoparticles. **(D)** N_2_ adsorption-desorption isotherms of ZIF-90 and MTZ. **(E)** UV-vis spectra of ZIF-90, TK-CPT, 2-ME, and MTZ.

The TK-CPT (prodrug of camptothecine) was synthesized according to the previously reported method (Figure S1) 22. The synthesized TK-CPT prodrug was characterized by nuclear magnetic resonance (NMR) and high-resolution mass (HRMS) spectrometry (Figure S2 and Figure S3.). ZIF-90 was formed by Zn^2+^ and imidazolate-2-carboxyaldehyde (2-ICA) through coordination interactions. The TK-CPT and 2-ME inhibitor were encapsulated into the pores of ZIF-90 nanoparticles using a simple one-pot method. ZIF-90, TK-CPT@ZIF-90 (TZ) and 2-ME@ZIF-90 (MZ) nanoparticles were also synthesized by the same method using as control groups. X-ray diffraction (XRD) was employed to characterize the successfully synthesized ZIF-90. As shown in Figure S4, TZ, both MZ and MTZ nanoparticles exhibited similar peaks with pure ZIF-90 crystals, indicating that the crystal lattice was remained after the encapsulation process. To characterize the sizes and morphologies of the nanoparticles, transmission electron microscope (TEM) and scanning electron microscope (SEM) were performed. Figure 1A and Figure S5 indicated that all the ZIF-90, TZ, MZ and MTZ nanoparticles have regular polygonal shapes with distinct edges and corners, and the sizes remain as ~80 nm after encapsulation. Finally, the surface of MTZ nanoparticles was coated with a layer of 4T1 cancer cell membrane to obtain MTZ@C. After the coating, the surface of MTZ@C nanoparticles became smooth and the size increased to about 100 nm. The dynamic light scattering (DLS) results are consistent with the ones of TEM, and the size of the cancer cell membrane coated nanoparticles increased slightly (Figure 1B and S6). The zeta potential was further used to characterize the successfully constructed nanoparticles, with the results of -25.8 ± 3.1 mV, -19 ± 1.4 mV, 16.9 ± 0.5 mV, and 14.6 ± 0.3 mV, for 2-ME, TK-CPT, ZIF-90, and MTZ, respectively. Compared with MTZ particles (14.6 ± 0.3 mV), the zeta potential of MTZ@C decreased to -10.3 ± 0.4 mV (Figure 1C), suggesting the successful coating of cancer cell membranes. Then, the specific surface area of the nanoparticles was calculated by the Brunauer-Emmett-Teller (BET) method, and the average pore diameter was calculated by the Barrett, Joyner, and Halenda (BJH) method (Figure 1D and Figure S7). The average pore size of the ZIF-90 surface was calculated to be about 3 nm and the surface area of MTZ nanoparticles (68.0 m²/g) is significantly lower than that of ZIF-90 (851.4 m²/g), demonstrating that the drugs were successfully encapsulated into the pores of ZIF-90. The successful loading of TK-CPT and 2-ME was further verified through UV-visible (UV-vis) and Fourier transform infrared (FT-IR) spectra (Figure 1E, and Figure S8). The stability of MTZ nanoparticles in water was then evaluated. Figure S9 showed that the sizes of the nanoparticles had almost no change in six days, indicating the good stability of the nanoparticles.

### ATP triggered collapse of MTZ nanoparticles

**Figure 2:**
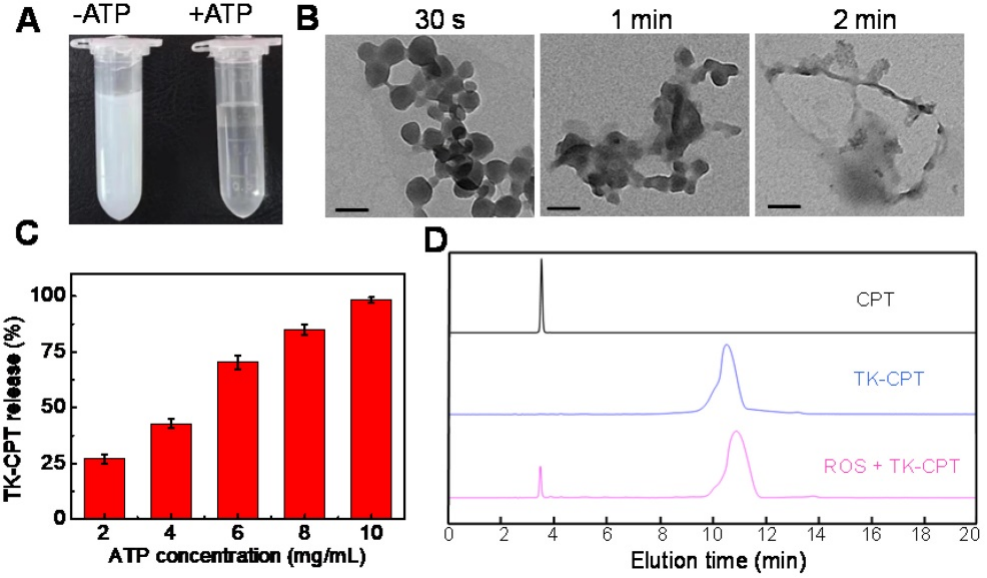
** (A)** Photograph of MTZ aqueous solution (5 mg/mL) without (left) or with (right) ATP (10 mg/mL) within 5 min. **(B)** TEM images of MTZ (0.5 mg/mL) at different intervals (30 s, 1 min and 2 min) after the addition of ATP (1 mg/mL). Scale bars are 100 nm. **(C)** Release rate of TK-CPT from the ZIF-90 framework (5 mg/mL) under different ATP conditions within 5 min. **(D)** HPLC analysis of TK-CPT upon 24 h incubation with O_2_^·-^.

The previous study has indicated that ZIF-90 would collapse in presence of ATP due to the stronger coordination interaction between ATP and Zn^2+^ than that between ICA and Zn^2+^. To verify this process, the MTZ nanoparticles were incubated with ATP. As shown in Figure 2A, the MTZ aqueous solution was turbid, which became clear in a short time after enough ATP was added for co-incubation. The results confirmed that the ZIF-90 framework could collapse in the presence of ATP. Zeta potential of MTZ in the absence and presence of ATP were measured, as shown in Figure S10, the zeta potential value of MTZ was 14.6 ± 0.3 mV, while the value changed to negative (-26.8 ± 0.6 mV) from positive when ATP was co-incubated with MTZ. The change of the zeta potential from positive to negative was considered because of the collapse of the ZIF-90 frame and the release of the drugs. To further confirm the collapse of ZIF-90 induced by ATP, MTZ nanoparticles (1 mg/mL) were treated with ATP (2 mM) for different time (30 s, 1 min, 2 min). TEM images of the above samples are shown in Figure 2B, which indicating that the MTZ nanoparticles were completely disintegrated in two minutes. To verify that the nanosystem was indeed triggered by ATP, the influence of pH on the stability of ZIF-90 was evaluated. As shown in Figure S11, no obvious change was observed for the degradation rate of MTZ nanoparticles under pH values ranging from 4.3 to 8.0, indicating that MTZ nanoparticles are stable in the physiological pH range.

### Drug-loading/releasing behaviour of MTZ nanoparticles

Then the loading efficiency of drug in ZIF-90 was measured by the fluorescence standard curves of TK-CPT and 2-ME (Figure S12). The loading amount of TK-CPT and 2-ME were calculated to be 23.1% (w/w) and 29.7% (w/w), respectively. Releasing rates of TK-CPT from the ZIF-90 framework under different ATP concentrations were further studied. As the concentration of ATP increased, the amount of released drug also gradually increased (Figure 2C). The drug would be released completely within a short time (< 5 min) owing to the quick reaction, which is consistent with Figure 2B (vide supra). The results demonstrated that the MTZ nanoparticles have excellent ATP-responsive releasing behavior. On the other hand, the thioketal linkers in TK-CPT are expected to respond to ROS and release parent CPT drug. To validate that ROS could efficiently induce the CPT release, KO_2_ was dissolved in anhydrous DMSO to generate superoxide anion (O_2_^-^), and further used to trigger the break of thioketal structure. As monitored by high performance liquid chromatography (HPLC), a new peak ascribed to CPT was observed except the one of TK-CPT in the presence of ROS (1 mM) upon 24 h incubation with TK-CPT, indicating the successful ROS-induced CPT release (Figure 2D).

### Endocytosis mechanism and intracellular ATP degradation behaviour of MTZ

Endocytosis inhibitors, CPZ (inhibitor of clathrin-mediated endocytsis) and EIPA (inhibitor of macropinocytosis), were incubated with 4T1 cells to test the cellular uptake pattern of nanoparticles. The cells without inhibitor were also incubated with MTZ@C at 4 °C to study the effect of energy. As shown in Figure S13, the uptake of MTZ@C was obviously inhibited for the cells treated with CPZ and low-temperature. The results indicated that the prepared nanoparticles entered the cells mainly through clathrin-mediated endocytosis and energy-dependent cellular endocytosis.

Next, the ATP-triggered drug release behaviors of nanomaterials in cancer cells and normal cells were examined. MTZ nanoparticles were incubated with 4T1 and TC-1 cells for 2 h and observed using CLSM images. As shown in Figure S14, the fluorescence intensity of TK-CPT in 4T1 cells was much higher than that in TC-1 cells. The results indicated that it is difficult to degrade MTZ nanoparticles and release internal drugs in normal cells (TC-1) because of the low ATP level.

### Mitochondria co-localization and ROS level monitoring

**Figure 3:**
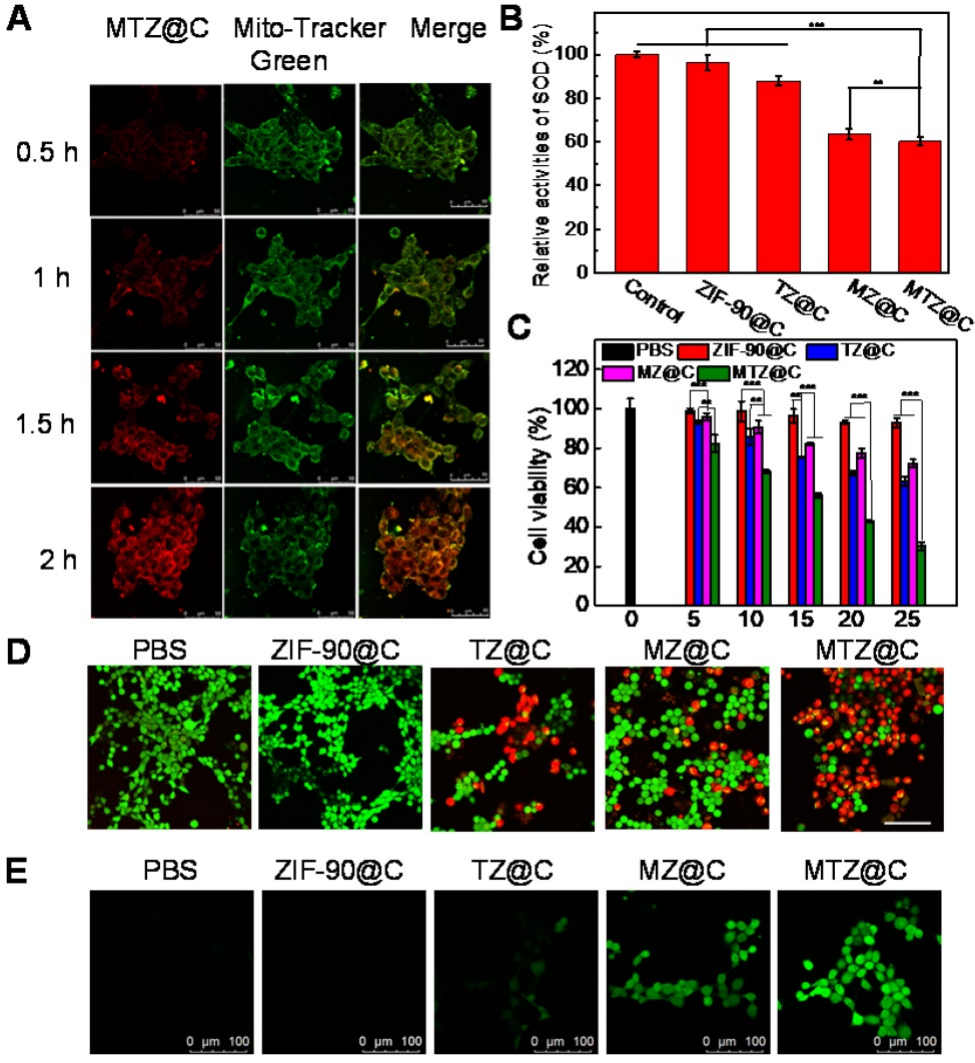
** (A)** Mitochondrial co-location images of MTZ@C nanoparticles incubated with 4T1 cells for different time. The cells were incubated with the probe mito-tracker green before confocal imaging. **(B)** The relative activity level of SOD in cells after incubation with different nanoparticles. Data were presented as the mean ± SD (n = 3), **P < 0.01, ***P < 0.001. **(C)** MTT assay of 4T1 cells with different treatments. Data were presented as the mean ± SD (n = 3), **P < 0.01, ***P < 0.001. **(D)** Confocal images of calcium AM and PI stained 4T1 cells after different treatments. Scale bars are 100 µm. **(E)** Confocal images of 4T1 cells after treated with PBS, ZIF-90@C, TZ@C, MZ@C, and MTZ@C. The cells were incubated with the probe DCFH-DA before confocal imaging.

Mitochondria is the main energy supply site for cells, and mitochondrial damage can activate programmed death of cancer cells. To test the mitochondria-targeting ability of MTZ@C nanoparticles, 4T1 cells were incubated with MTZ@C nanoparticles and stained with the commercial mitochondria dye mito-tracker green. CLSM imaging showed that the red fluorescence in 4T1 tumor cells gradually increased with the prolonged incubation time, implying that the accumulation of MTZ@C in mitochondria is gradually increasing (Figure 3A). According to the quantified fluorescence results, the nanoparticle accumulation in mitochondria gradually increased after incubating MTZ@C with 4T1 cells for 2 h (Figure S15). In the mitochondria co-localization experiment, red fluorescence and green fluorescence have a good overlap, and the Pearson correlation coefficients *R* = 0.79 (Figure S16). Besides, the bio-TEM images also confirmed that MTZ@C nanoparticles were efficiently enter the mitochondria (Figure S17). All the above experimental results showed that the positively charged ZIF-90 mainly localized in mitochondria. To check the SOD activity inhibiting capability of 2-ME, a commercially available kit (Total Superoxide Dismutase Assay Kit with WST-8) was used to measure the viability of SOD in 4T1 cells. As displayed in Figure 3B, the groups containing 2-ME inhibitor (MZ@C, MTZ@C) had a significant inhibitory effect on SOD compared with other control groups (PBS, ZIF-90@C, TZ@C), with the relative activity of SOD being reduced about 47.5% and 49.8%. It is well-known that SOD can scavenge superoxide anion (O_2_^·-^) in cells 24. As a result, when MTZ@C nanoparticles were co-incubated with 4T1 cells, 2-ME could inhibit the activity of SOD and increase the level of ROS in cells. To estimate the level of ROS in various groups, DCFH-DA was selected as an intracellular ROS fluorescent probe. Figure 3E clearly showed that the control groups (PBS, ZIF-90@C, TZ@C) produced weaker fluorescence signals than the groups containing 2-ME, implying that 2-ME effectively induced a higher level of ROS in cells. These results demonstrated that the ZIF-90 based platform could target mitochondria and induce the up-regulation of ROS.

### Cytotoxicity assay

The therapeutic effect of MTZ@C nanoparticles against cancer cells was confirmed via an MTT assay. As shown in Figure 3C, the cell viability of TZ@C and MZ@C groups was 65.1% and 72.1%. While the viability of 4T1 cells was reduced to about 30.4% when incubated with MTZ@C, indicating TK-CPT combined with 2-ME had excellent anticancer effects. Moreover, the viability of cells incubated with ZIF-90@C was about 93.1%, confirming the safety of ZIF-90 as a biological carrier. The fluorescence images of dead cells (red) and living cells (green) stained with PI and Calcein-AM after different treatments were shown in Figure 3D and Figure S18. After incubated with cells for 10 h, only green fluorescence was observed in ZIF-90@C group, indicating that no apoptosis occurred due to the good biocompatibility of ZIF-90. The florescence images of TZ@C and MZ@C groups showed that a minor portion of 4T1 cells dead, while the strongest red fluorescence could be observed for MTZ@C group, because of the 4T1 cell apoptosis and decreased cell viability. Similar results were achieved from the cell apoptosis analysis using flow cytometry (Figure S19). The apoptosis-associated proteins in 4T1 cells were further detected by western blotting analysis. After incubated with nanoparticles, the expression of cytochrome c and caspase-3 were significantly increased for the groups of TZ@C, MZ@C and MTZ@C, while the most pronounced increase was observed for the MTZ@C group (Figure S20). Western blotting results confirmed that the mitochondrial damage caused by MTZ@C can activate the programmed death of cancer cells.

To confirm the important effect of ATP on the degradation of intracellular MTZ@C nanomaterials, 4T1 cells were pretreated with sodium azide (NaN_3_, 10 mM) for 12 h for decreasing the amount of intracellular ATP in advance. The cytotoxicity assay showed that NaN_3_ and MTZ@C co-treated cells showed higher cell viability than that treated using only MTZ@C. The results indicated that ATP can effectively degrade MTZ@C to release chemotherapeutic drugs and enhance the cytotoxicity to cells (Figure S21).

### Validation of membrane targeting of cancer cells

To verify that homologous cell membranes have good targeting effects, we co-incubated MTZ and MTZ@C nanoparticles (MTZ coated with 4T1 cell membrane) with 4T1 cells. After incubated the nanoparticles with cells for 2 h, the stronger fluorescence intensity was observed for the group of MTZ@C co-incubated with 4T1 cells, while the fluorescence for the group of MTZ was much weaker (Figure S22). The results suggested that nanoparticles encapsulated with homologous cell membranes are easier to endocytose by tumor cells.

The targeting ability of cancer cell membrane coated nanoparticles was further evaluated *in vivo* by intravenous injection of MTZ and MTZ@C into 4T1 tumor mice. Compared with MTZ, the membranes-encapsulated MTZ@C showed more significant tumor targeting effect. According to the *in vivo* imaging results, MTZ@C has a maximum tumor accumulation at 24 h post-injection (Figure S23). After 24 h injection, the tumor, major organs, and metabolites of the mice were collected to separately measure the concentrations of Zn^2+^ using inductively coupled plasma atomic emission spectrometry (ICP-AES). As shown in Figure S24, the concentration of Zn^2+^ in tumor for MTZ@C group was significantly higher than that of the MTZ group, indicating that the targeting ability of MTZ@C was much better than MTZ nanoparticles. However, the concentration of Zn^2+^ in urine (0.275 µg/mL) and feces (0.39 µg/mg) of the MTZ group was significantly higher than that of the MTZ@C group (urine: 0.078 µg/mL, feces: 0.26 µg/mg), demonstrating that MTZ nanoparticles were mainly metabolized out of the body due to the lack of cancer cell membrane coating.

### *In vivo* therapeutic effect of ZIF-90 based nanoplatform

**Figure 4:**
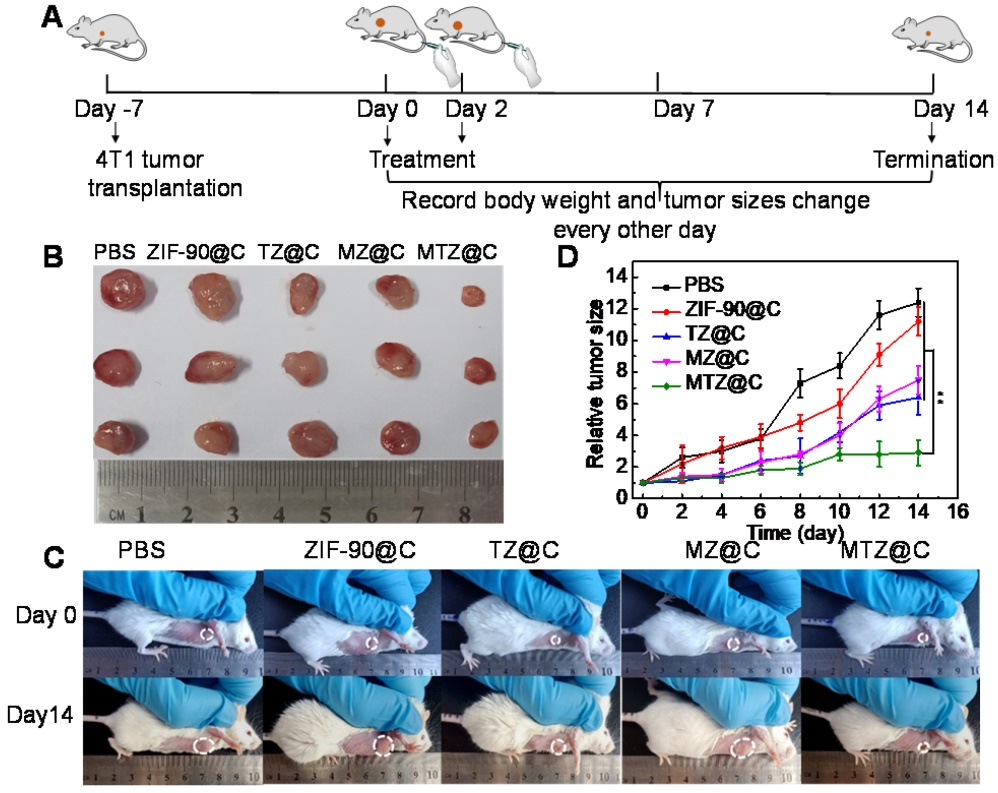
** (A)** Diagrammatic representation of the therapeutic experiment. **(B)** Photographs of dissected tumors after the mice were treated with different materials for 14 days. **(C)** Photographs of the mice taken before treatment (0 days) and at the 14^th^ day with different treatments. **(D)** Tumor growth curves within 14 days after the mice were received different treatments. Data were presented as the mean ± SD (n = 5), **P < 0.01.

Encouraged by the excellent anticancer efficacy of MTZ@C *in vitro*, the therapeutic effect of nanoparticles *in vivo* was evaluated in a mouse model. Figure 4A shows the details of the treatment process. During 14 days after the first treatment, the tumor sizes and body weights were recorded every two days. After injection of different nanoparticles (saline, ZIF-90@C, TZ@C, MZ@C, and MTZ@C), the weight of mice in each group had no obvious loss (Figure S25). In addition, the blood parameters (Figure S26) and standard haematology markers (Figure S27) of the mice with different treatments showed no significant differences, demonstrating the negligible inflammation and the low hepatic and kidney toxicity of the nano-materials. Moreover, the hematoxylin & eosin (H&E) stained images of major tissues, including heart, liver, spleen, lung, and kidney, showed no significant morphological changes (Figure S28), illustrating that the nanoparticles had the desirable biocompatibility and biosafety for the application of tumor chemotherapy. As can be seen from Figure 4B, the tumor in the group treated with MTZ@C was significantly smaller than that in the control groups. The dissected tumor pictures also confirmed that the tumor volume in the MTZ@C treated group is the smallest (Figure 4C, 4D, and Figure S29). The above experimental results indicated that the designed nanoparticles exhibited a good therapeutic effect on solid tumors. The H&E and TUNEL stained images of tumor tissue (Figure S30 and Figure S31) showed that the groups treated with saline and ZIF-90@C did not have obvious tumor cell apoptosis, while the groups treated with TZ@C, MZ@C, and MTZ@C had tumor cell apoptosis. The highest level of tumor cells apoptosis and necrosis was observed for the group treated with MTZ@C, indicating that the ZIF-90 based strategy inducing mitochondrial cascade reactions had a remarkable synergistic therapy effect.

## Conclusions

In summary, we demonstrate an ATP-triggered platform based on nanoscale ZIF-90 to induce mitochondrial cascade reactions for cancer therapy. The nanoplatform was synthesized using a simple one-pot method with CPT prodrug and 2-ME. Then the homologous cancer cell membrane was coated on the surface, endowing the nanoparticles capability to target cancer cells. The positive charges on the surface of ZIF-90 enable it to actively target mitochondria via the electrostatic interaction. In the presence of ATP, ZIF-90 can quickly disintegrate in mitochondria and liberate loaded drugs. 2-ME can inhibit SOD activity and up-regulate the level of ROS, leading to the high oxidative stress in mitochondria. The thioketal linkers in CPT prodrug can respond to increased ROS and release parent CPT drug. Through this cascade of reactions, the ZIF-90 based platform can effectively inhibit the growth of cancer cells and tumors. *In vitro* and *in vivo* experiments indicated that the designed nanoplatform exhibited low cytotoxicity, good mitochondria-targeting ability, and excellent therapeutic effect. We anticipate that this ATP-triggered method to induce mitochondrial biochemical reactions can provide new insights for designing organelle-targeted therapeutic platforms.

## Supplementary Material

Supplementary methods and figures.

## Abbreviations

ZIF-90: zeolitic imidazole framework-90
CPT: camptothecin
TK-CPT: thioketal-linked camptothecin
2-ME: 2-Methoxyestradiol
SOD: superoxide dismutase
ROS: reactive oxygen species
ATP: adenosine triphosphate
NMR: Nuclear magnetic resonance
MS: mass spectrometry
2-ICA: imidazolate-2-carboxyaldehyde
XRD: X-ray diffraction
TEM: Transmission electron microscope
SEM: scanning electron microscope
DLS: dynamic light scattering
O_2_^-^: superoxide anion
HPLC: high performance liquid chromatography
CLSM: Confocal laser scanning microscopy
DCFH-DA: 2',7'-dichlorofluorescin diacetate
ICP-AES: inductively coupled plasma atomic emission spectrometry
