# Supplementary material for: ATP-triggered mitochondrial cascade reactions for cancer therapy with nanoscale zeolitic imidazole framework-90
Source: Theranostics. 2021 Jun 26;11(16):7869–78. doi: 10.7150/thno.59593 (PMC8315074; doi:10.7150/thno.59593)
Supplement: Supplementary file 1 — Supplementary methods and figures. [file thnov11p7869s1.pdf]

Supporting Information

ATP-triggered mitochondrial cascade reactions for cancer therapy with nanoscale zeolitic imidazole framework-90

Wei Pan,[†✉] Bingjie Cui,[†] Kaiye Wang, Mingwan Shi, Fei Lu, Na Li,[✉] and Bo Tang[✉]

Experimental Section

Reagents and materials.

Trifluoroacetic acid (TFA) and 2,2-Bis(hydroxymethyl)propionic acid (DMPA) were purchased from Aladdin Reagent Co., Ltd.; Carbodiimide (EDC) was purchased from Alfa Esha Chemical Co., Ltd.; 3-mercaptopropionic acid, Imidazole-2-carboxaldehyde (ICA), trioctylamine (TOA), 2-Methoxyestradiol (2-ME), and Camptothecin (CPT) were purchased from Saen Chemical Technology, Co., Ltd.; 2′, 7′-dichlorofluorescein diacetate (DCFH-DA) and total SOD activity detection kit (wst-8 method) were obtained from Beyotime Biotechnology (China); 3-(4,5-dimethyl-thiazol-2-yl)-2,5-diphenyltetrazolium bromide (MTT), propidium iodide (PI) and calcein acetoxymethyl ester (Calcein AM) were purchased from Sigma-Aldrich (USA); Mouse breast cancer cell line (4T1) was purchased from Shanghai AOLU Biological Technology Co. Ltd. (China); Mouse lung epithelial cell line (TC-1) was purchased from KeyGEN biotechnology Company (Nanjing, China).

Apparatus

Transmission electron microscopy (TEM, HT7700, Japan) was carried out to characterize the morphology of the nanoparticles. Zeta potential was performed on a Malvern Zeta Sizer Nano (Malvern Instruments). Homogenizer (IKA, Germany, T10 basic ultra-turrrax). X-ray powder diffractometer (Bruker D8, Germany). Fourier transform infrared spectroscopy (FT-IR) was collected on a Nicolet Impact 410 FTIR spectrometer. UV-Vis spectrophotometer (UV-1700, Shimadzu, Japan) was used to measure the UV-Vis absorption spectra. Fluorescence spectrometer (FLS980, Edinburgh Instruments Ltd., UK) was used to detect the fluorescence of nanoparticles. pH values were measured by a pH-3c digital pH-meter (Shanghai LeiCi, China). Confocal laser scanning microscope (LEICA TCS SP5, Germany) and live animal imaging system (IVIS Lumina

III, US) were applied in vitro cell experiments and *in vivo* imaging, respectively. MTT microplate reader (Rayto RT-6000, US) and inductively coupled plasma atomic emission spectrometer (ICP-AES, ICAP 7600, Thermo Fisher, US) were used in this work.

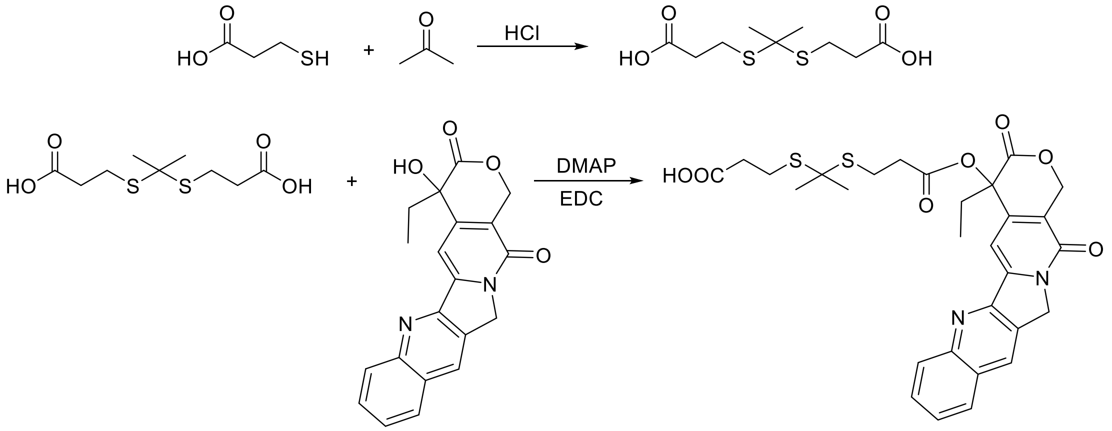

Figure S1. Synthetic procedure employed for ROS-responsive prodrug (TK-CPT).

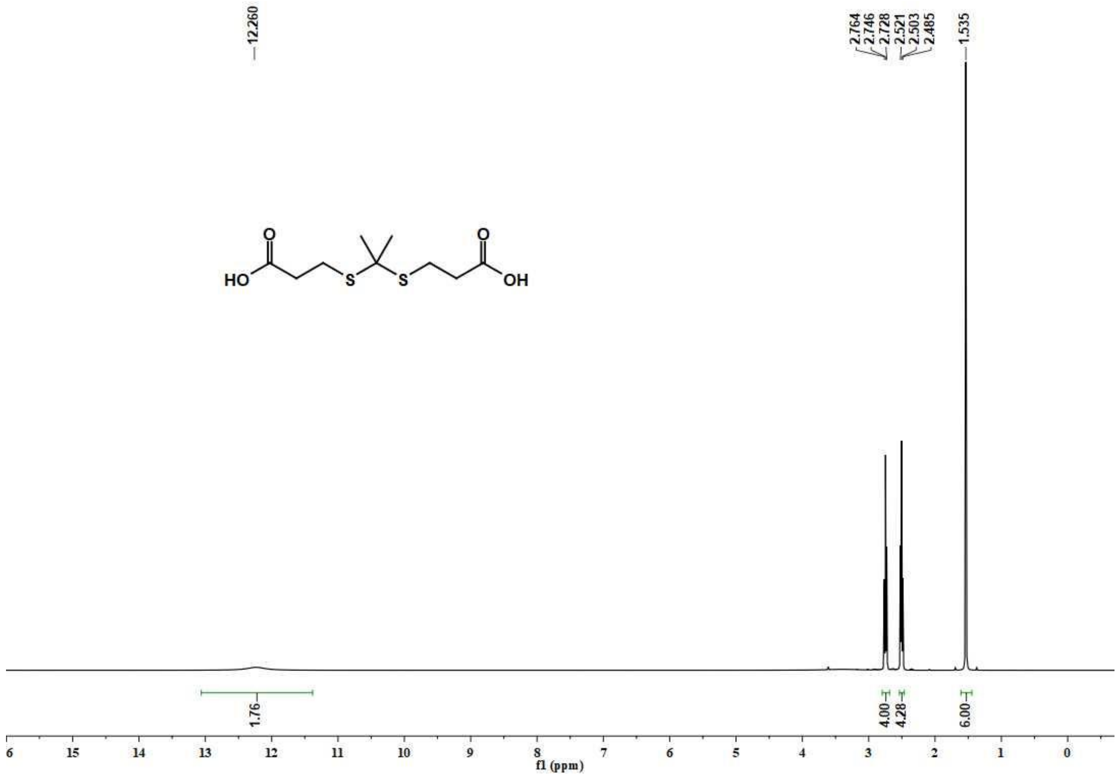

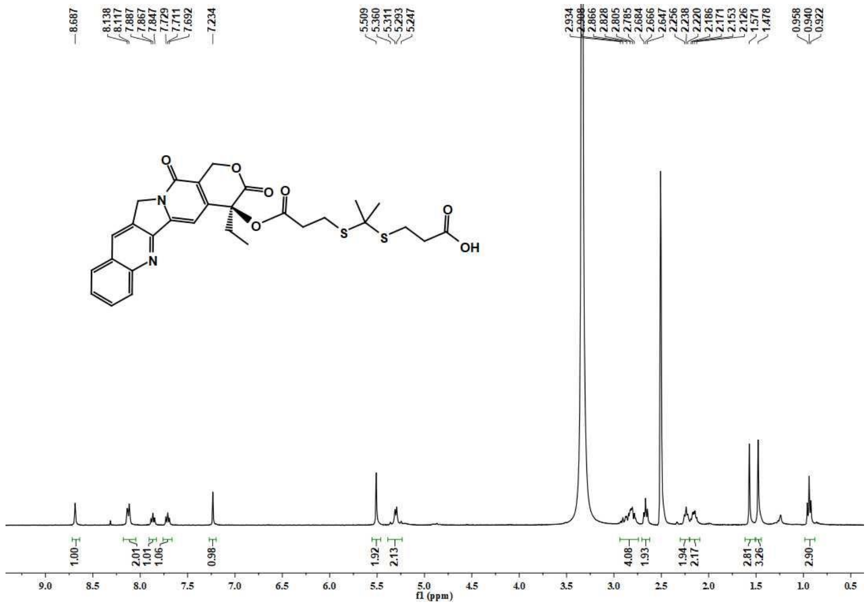

Figure S2. ^{1}H NMR data of TK and TK-CPT.

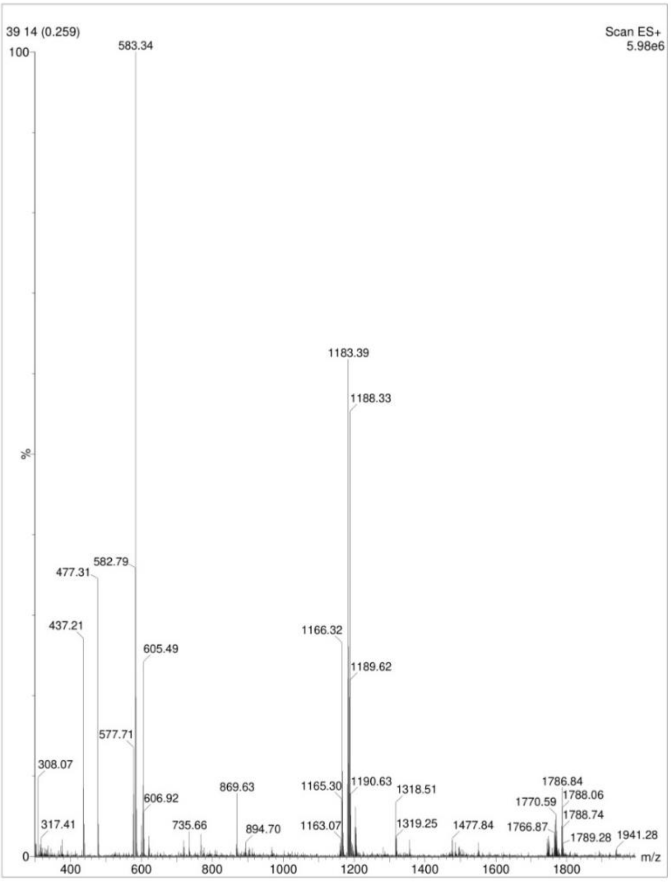

Figure S3. High-resolution mass data of TK-CPT.

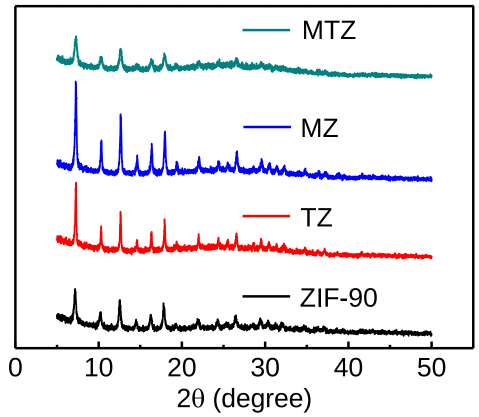

Figure S4. X-ray diffraction (XRD) diffractograms of ZIF-90, TZ, MZ and MTZ.

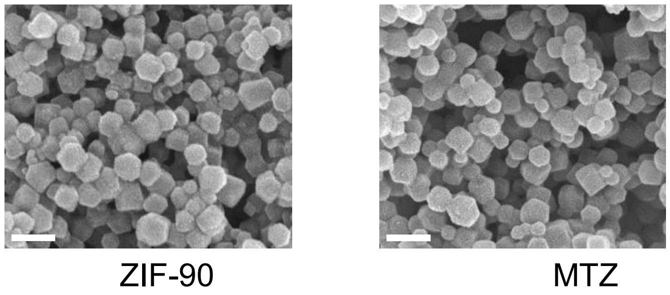

Figure S5. SEM images of ZIF-90, MTZ. Scale bars are 100 nm.

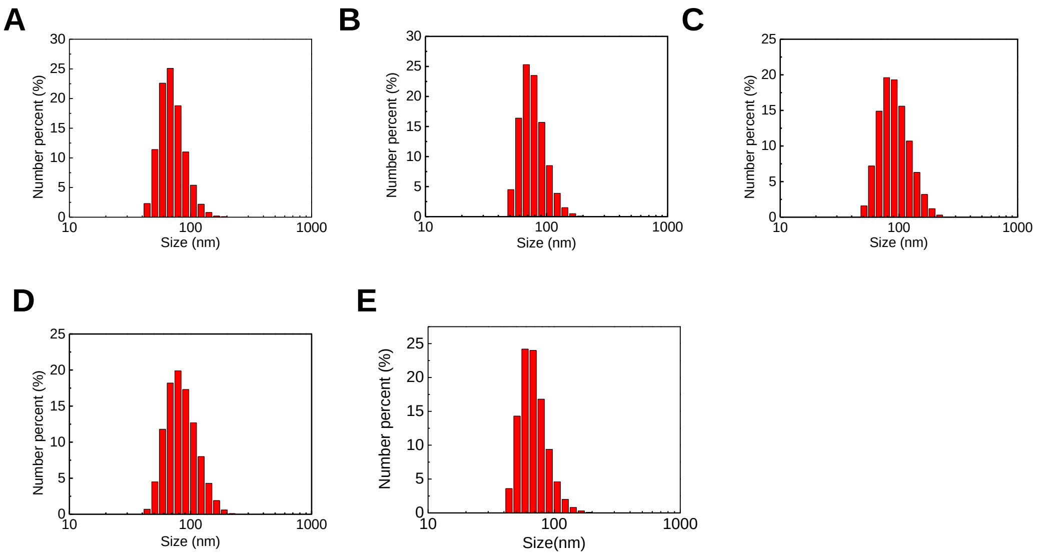

Figure S6. Size distributions of different nanoparticles: (A) ZIF-90, (B) MZ, (C) TZ, (D) MTZ, (E) MTZ@C.

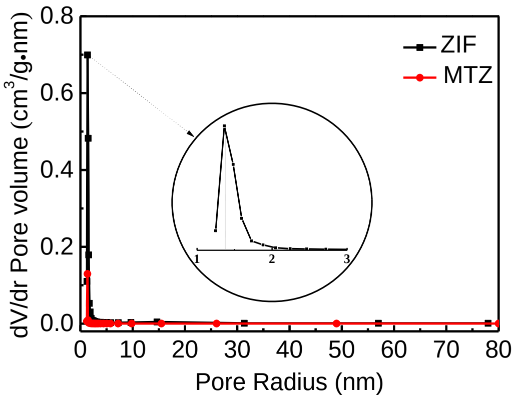

Figure S7. Pore width distributions of ZIF-90 and MTZ.

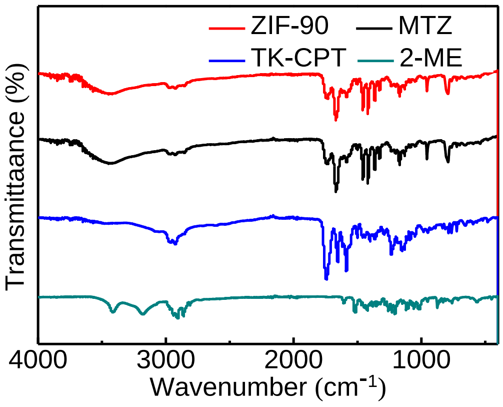

Figure S8. FT-IR spectra of ZIF-90, TK-CPT, 2-ME and MTZ.

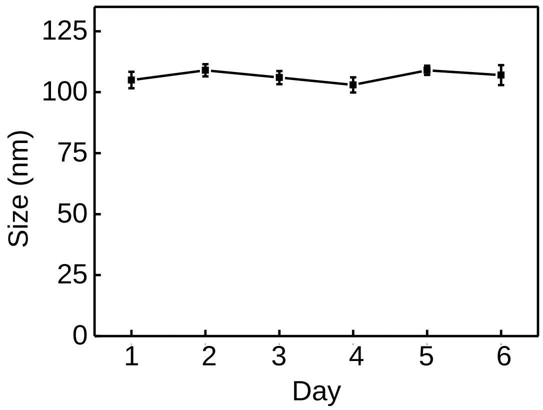

Figure S9. The sizes of MTZ nanoparticles in aqueous solution measured by DLS within 6 days.

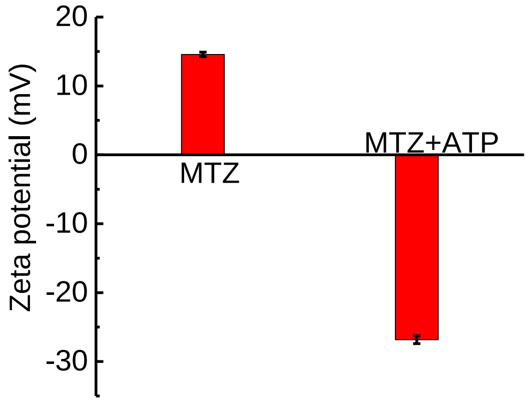

Figure S10. The zeta potential of MTZ nanoparticles before and after adding ATP.

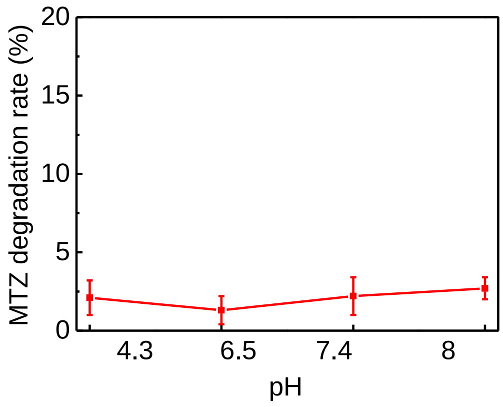

Figure S11. Degradation rate of MTZ nanoparticles in aqueous solutions with different pH values.

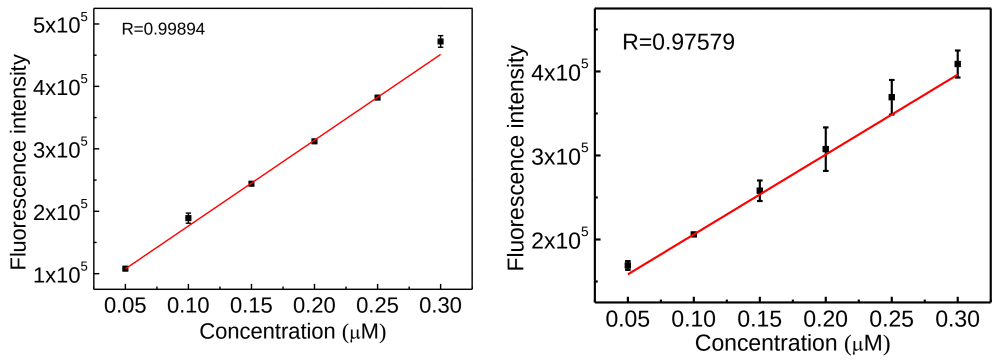

Figure S12. The quantification of TK-CPT (left) and 2-ME (right) from the fluorescence spectra.

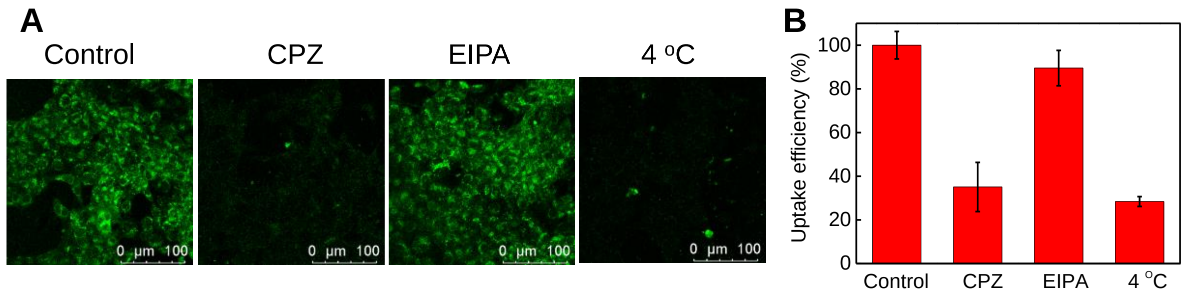

Figure S13. (A) Cellular uptake of MTZ@C by 4T1 cells treated with CPZ (inhibitor of clathrin-mediated endocytsis) or EIPA (inhibitor of macropinocytosis) for 1 h at 37 °C, or pre-incubated for 2 h at 4 °C (energy inhibitor). (B) Relative fluorescence intensities of TK-CPT from MTZ@C internalized by 4T1 cells in Figure S13(A).

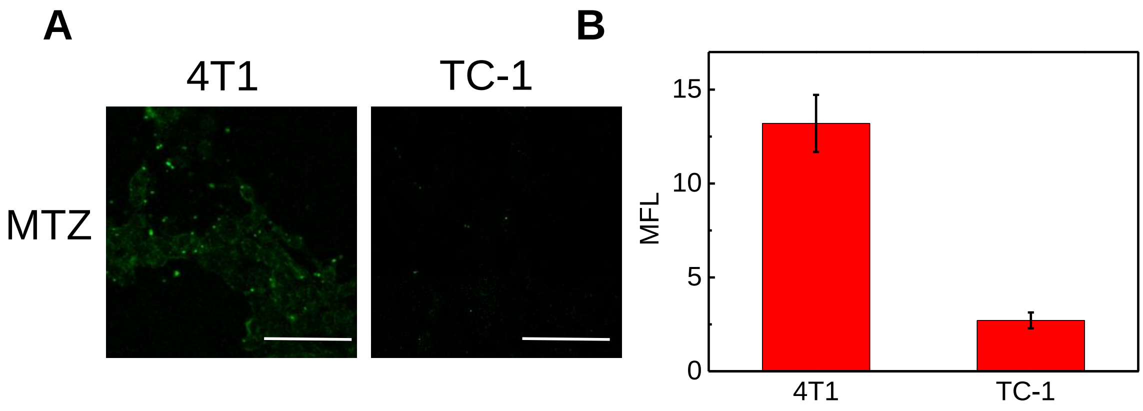

Figure S14. (A) Confocal images of 4T1 and TC-1 cells after incubated them with MTZ nanoparticles for 2 h to investigate the ability of intracellular ATP to degrade MTZ. The scale bars are 100 μm. (B) Relative fluorescence intensities of free TK-CPT released from MTZ internalized by 4T1 and TC-1 cells in Figure S14(A).

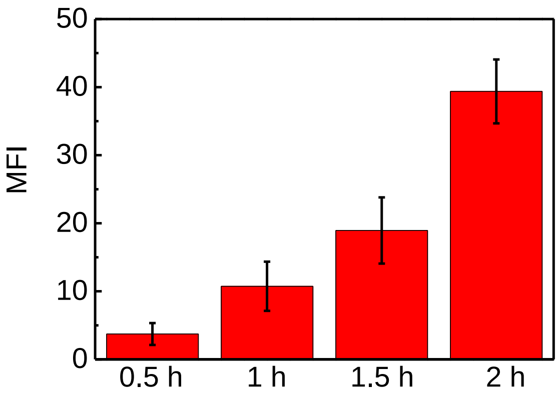

Figure S15. The relative fluorescence intensities of TK-CPT produced from MTZ@C in mitochondria, after incubated MTZ@C and 4T1 cells for different times (0.5 h, 1 h, 1.5 h, 2 h).

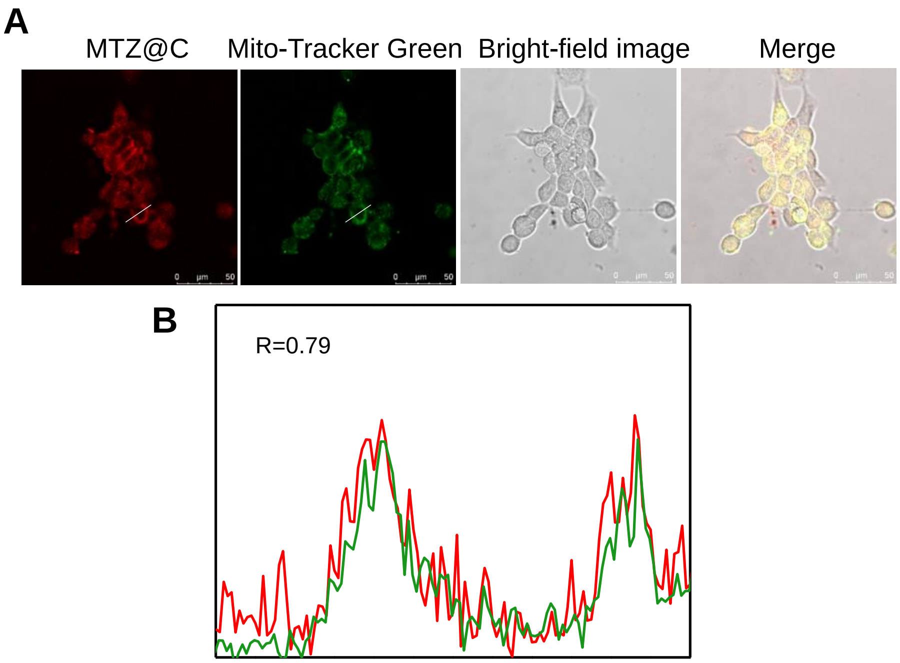

Figure S16. (A) Confocal images of subcellular colocalization of MTZ@C (25 μg/mL) in 4T1 cancer cells after incubated for 2 h. (B) Intensity profiles along the line segment in images. Red and green lines represent the intensities of TK-CPT and MitoTracker, respectively.

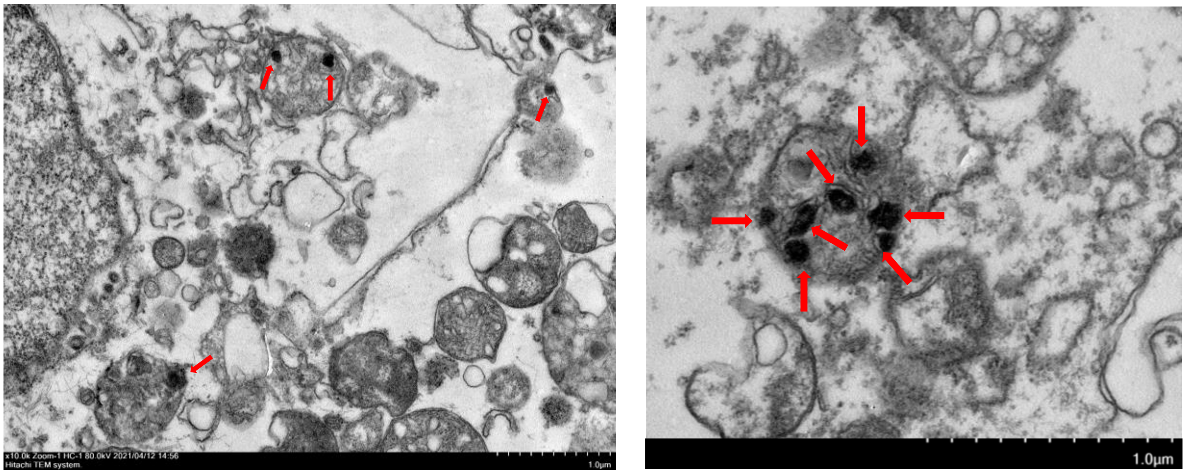

Figure S17. Bio-TEM images of 4T1 cells after incubation with MTZ@C for 2 h. The red arrows in the images show the nanoparticles in mitochondria.

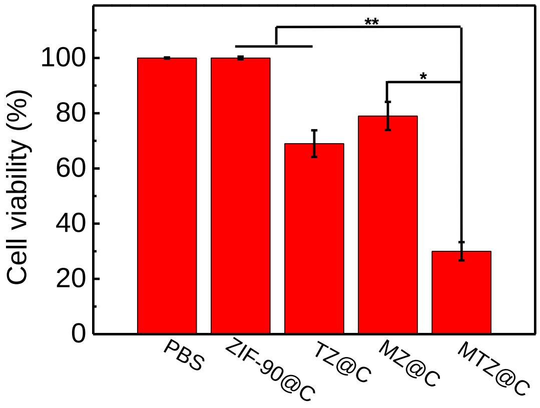

Figure S18. Results of AM and PI staining 4T1 cell survival statistics. Data were presented as the mean ± SD (n = 3), *P < 0.05, ***P < 0.01.

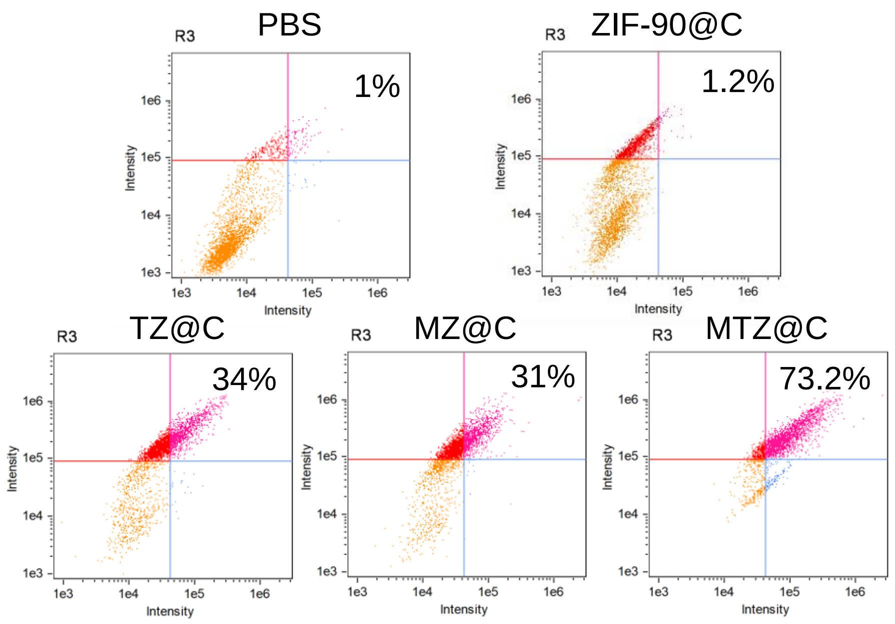

Figure S19. Flow cytometric analysis of apoptosis based on Annexin V-FITC (horizontal axis)/7-AAD (vertical axis). The lower left, lower right, upper right, and upper left regions indicate healthy cells, early apoptotic cells, late apoptotic cells, and necrotic cells, respectively. The numerical values indicate the percentage of the corresponding region.

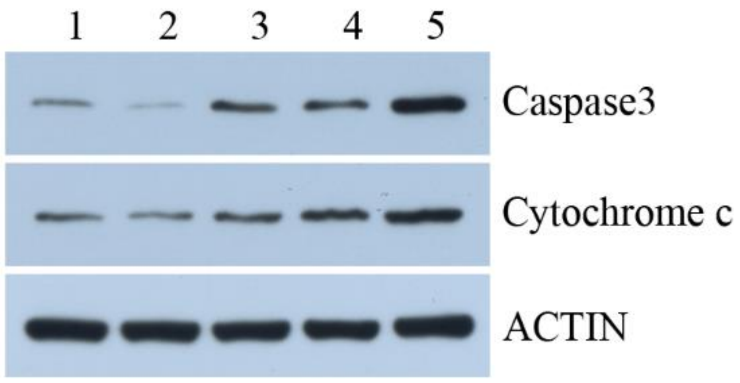

Figure S20. Western blotting analysis for the expression of Cyto C and cleaved-caspase 3 proteins after different treatments (1: PBS, 2: ZIF-90@C, 3: MZ@C, 4: TZ@C, 5: MTZ@C) for 10 h. Actin was used as a control.

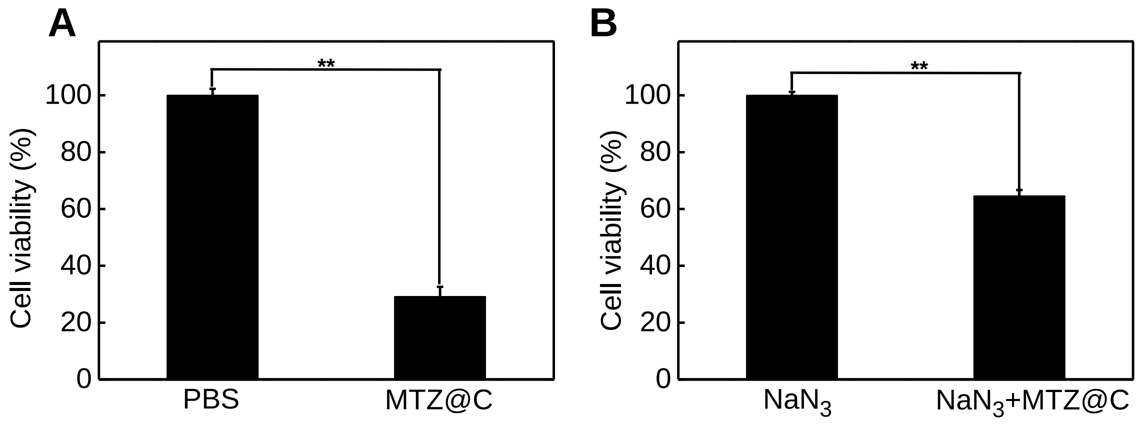

Figure S21. (A) Toxicity of MTZ@C nanoparticles to cells without NaN_3 pretreatment. (B) Toxicity of MTZ@C nanoparticles to cells after being pretreated with NaN_3 for 12 h. Data were presented as the mean ± SD (n = 3), **P < 0.01.

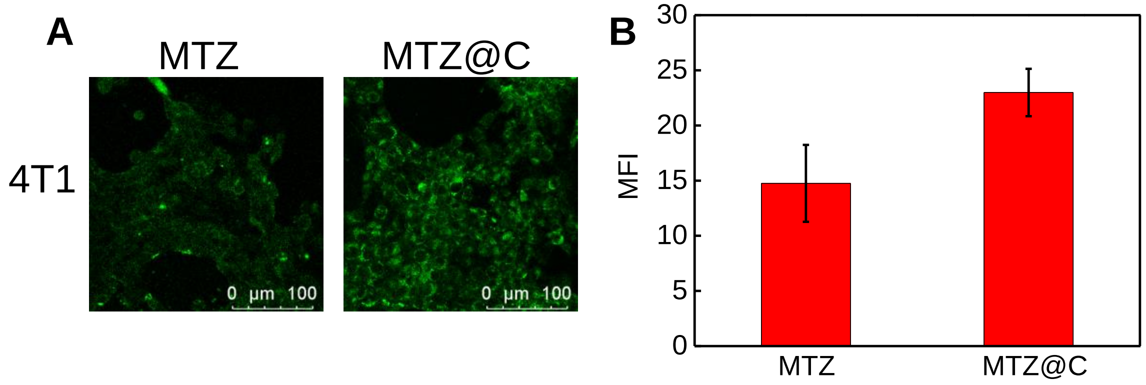

Figure S22. (A) Confocal fluorescence images of 4T1 cells after incubated them with the TK-CPT loaded MTZ and MTZ@C nanoparticles for 2 h. (B) Relative fluorescence intensities of TK-CPT loaded MTZ and MTZ@C nanoparticles were incubated with 4T1 cells in Figure S22(A).

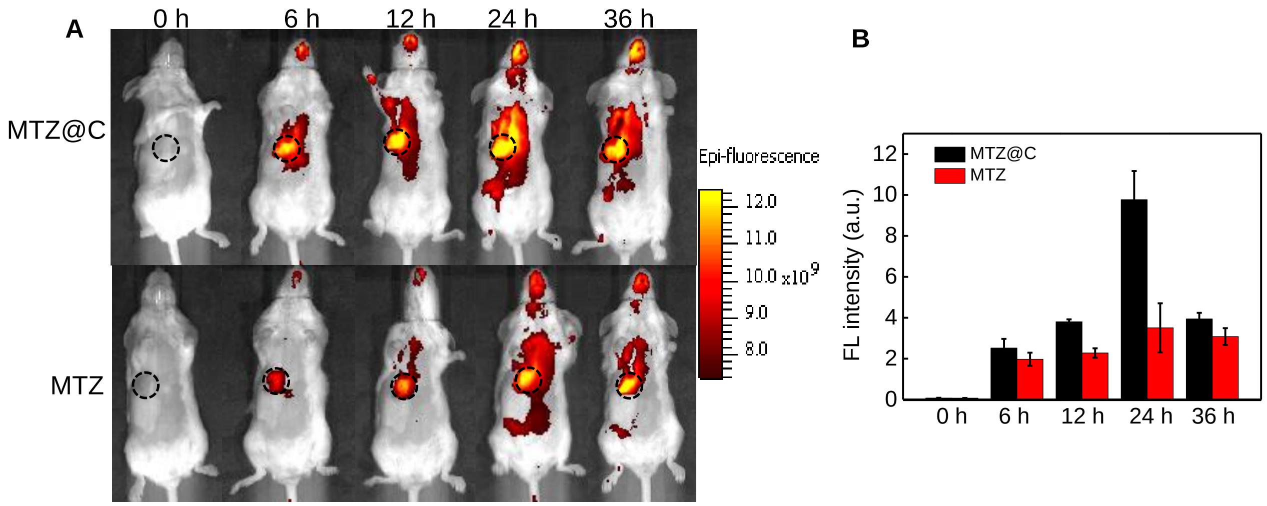

Figure S23. (A) *In vivo* fluorescent images of the tumor-bearing mice after intravenous injection of MTZ or MTZ@C at different times (0 h, 6 h, 12 h, 24 h, 36 h). (B) Relative fluorescence signal in tumor at different intervals of tumor bearing mice after intravenous injection of MTZ@C or MTZ. Data were presented as the mean ± SD (n = 3).

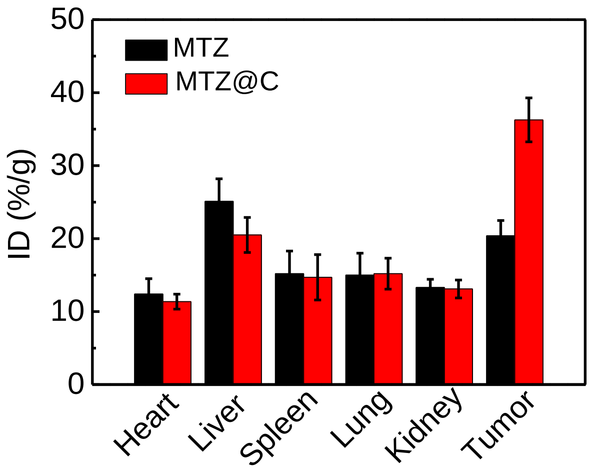

Figure S24. Concentration of Zn^{2+} in different organs and tumors after the mice were injected with MTZ@C or MTZ.

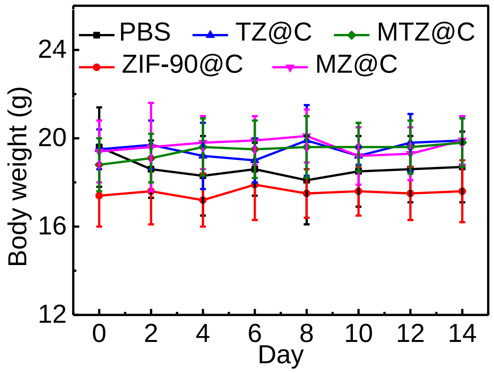

Figure S25. The mice body weight changes within 14 days after treatment. Data were presented as the mean ± SD (n = 5).

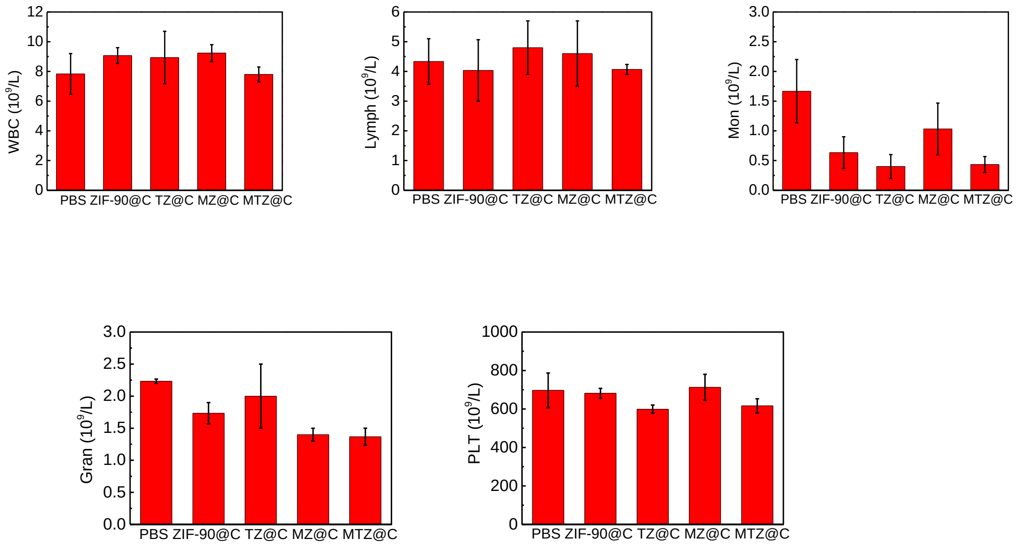

Figure S26. Haematological data of the mice intravenously injected with different samples at the 14th day post-injection. The terms are noted as followed: white blood cells (WBC), lymphocyte (Lymph), monocytes (Mon), neutrophils (Gran), and platelets (PLT).

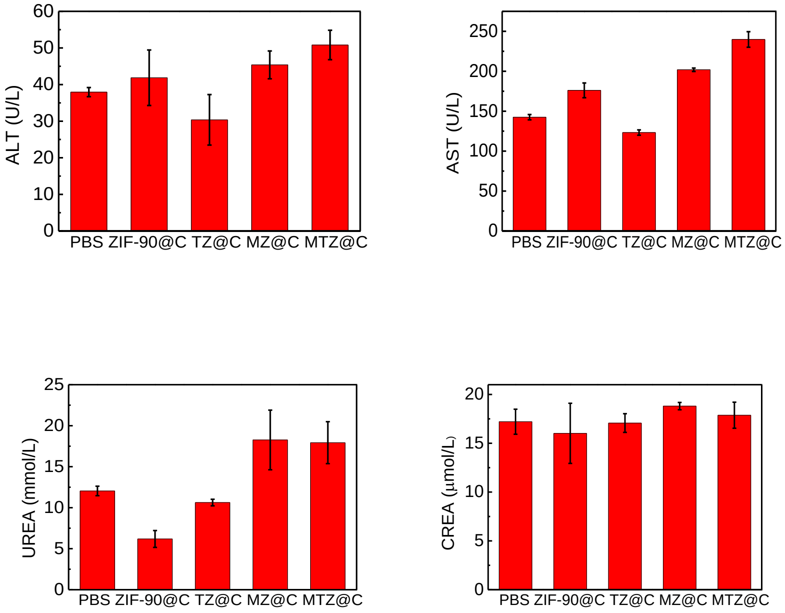

Figure S27. Blood biochemical analysis at the 14th day post-injection. The terms are following: alanine aminotransferase (ALT), aspartate aminotransferase (AST), UREA and creatinine (CREA).

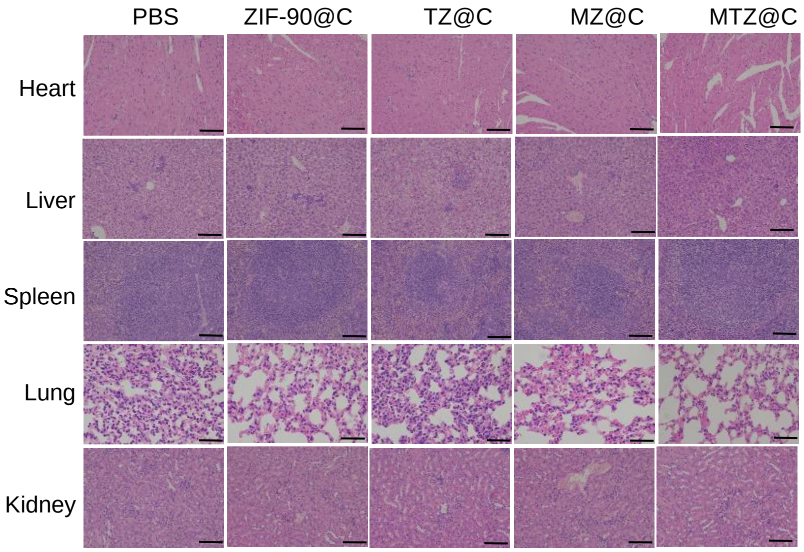

Figure S28. H&E staining of major organs (heart, liver, spleen, lung and kidney) after different treatments. Scale bars are 100 μm.

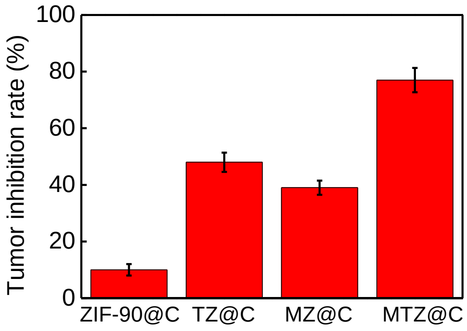

Figure S29. Tumor inhibition rate of tumor bearing mice with different treatments. The tumor inhibition rate was calculated by the formula: $(1 - V_{experiment}/V_{control}) \times 100\%$, using the group treated with PBS as a control. Data were presented as the mean ± SD (n = 5)

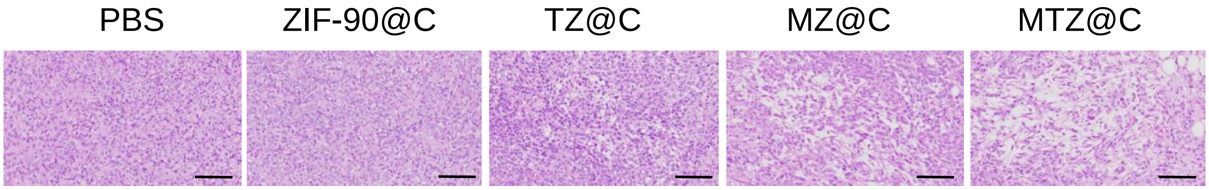

Figure S30. H&E staining images of tumor slides after different treatments. Scale bars are 100 μm.

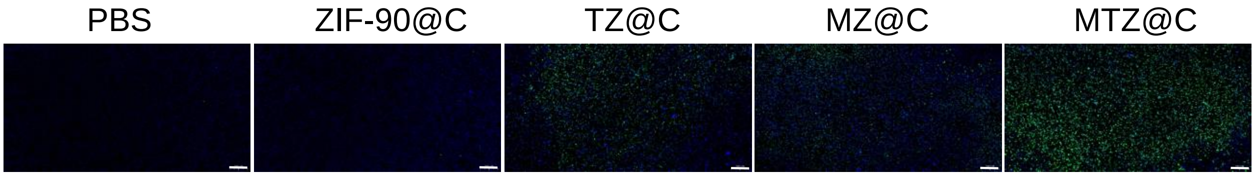

Figure S31. TUNEL staining images of tumor slides after different treatments. Scale bars are 100 μm.
